# A novel technique for solving unsteady three-dimensional brownian motion of a thin film nanofluid flow over a rotating surface

**DOI:** 10.1038/s41598-023-40410-3

**Published:** 2023-08-15

**Authors:** Payam Jalili, Ali Ahmadi Azar, Bahram Jalili, Davood Domiri Ganji

**Affiliations:** 1 Department of Mechanical Engineering, North Tehran Branch, Islamic Azad University, Tehran, Iran; 2 Department of Mechanical Engineering, Babol Noshirvani University of Technology, P.O. Box 484, Babol, Iran

**Keywords:** Mechanical engineering, Fluid dynamics

## Abstract

The motion of the fluid due to the swirling of a disk/sheet has many applications in engineering and industry. Investigating these types of problems is very difficult due to the non-linearity of the governing equations, especially when the governing equations are to be solved analytically. Time is also considered a challenge in problems, and times dependent problems are rare. This study aims to investigate the problem related to a transient rotating angled plate through two analytical techniques for the three-dimensional thin film nanomaterials flow. The geometry of research is a swirling sheet with a three-dimensional unsteady nanomaterial thin-film moment. The problem's governing equations of the conservation of mass, momentum, energy, and concentration are partial differential equations (PDEs). Solving PDEs, especially their analytical solution, is considered a serious challenge, but by using similar variables, they can be converted into ordinary differential equations (ODEs). The derived ODEs are still nonlinear, but it is possible to approximate them analytically with semi-analytical methods. This study transformed the governing PDEs into a set of nonlinear ODEs using appropriate similarity variables. The dimensionless parameters such as Prandtl number, Schmidt number, Brownian motion parameter, thermophoretic parameter, Nusselt, and Sherwood numbers are presented in ODEs, and the impact of these dimensionless parameters was considered in four cases. Every case that is considered in this problem was demonstrated with graphs. This study used modified AGM (Akbari–Ganji Method) and HAN (Hybrid analytical and numerical) methods to solve the ODEs, which are the novelty of the current study. The modified AGM is novel and has made the former AGM more complete. The second semi-analytical technique is the HAN method, and because it has been solved numerically in previous articles, this method has also been used. The new results were obtained using the modified AGM and HAN solutions. The validity of these two analytical solutions was proved when compared with the Runge–Kutta fourth-order (RK4) numerical solutions.

## Introduction

In science, especially chemistry, condensate production from a cooling and saturated vapor is very substantial. Many researchers investigated this phenomenon under various circumstances. Sparrow and Gregg^1^ analyzed film condensation on a rotating plate on pure saturated steam. The centrifugal field associated with the rotation moves the condensate outward along the disc's surface without requiring gravitational forces. In this problem, the governing equations were solved numerically, and finally, results were given for heat transfer and condensate layer thickness, torque, temperature, and velocity profiles. Beckett et al.^2^ investigated the problem of laminar condensation on a swirling disk in a large volume of static vapor for low and high cooling rates on the disk surface. The governing equations were converted into a set of ODEs using similarity transformation and solved numerically, and solutions were compared via previously published results. Chary and Sarma^3^ considered the problem of vapor-to-liquid transition in the presence of constant axial suction at a permeable condensing surface. The governing equations were reduced into a set of ODEs. The Runge–Kutta numerical method was used to calculate the heat transfer coefficient, and limiting solutions for very thin condensate films were obtained. They determined that the heat transfer coefficient can be increased to any desired level by correctly selecting the suction parameter value. Attia and Aboul-Hassan^4^ investigated the transient motion of a viscous conducting fluid due to the swirling of an infinite, non-conducting, porous disk with a uniform magnetic field and the Hall effect. The governing equations were solved numerically, and the solution showed that including injection or suction from the disk surface in addition to the Hall flow gives interesting results. Bachok et al.^5^ investigated the transient boundary layer of a nanofluid flow on a permeable stretching/shrinking sheet. The governing equations are reduced into nonlinear ODEs and solved numerically. Freidoonimehr et al.^6^ studied a nanofluid unsteady MHD laminar free convection flow on a perpendicular sheet. The governing equations are reduced into the system of ODEs by a suitable similarity transformation and solved numerically with the RK4 method. Makinde et al.^7^ investigated the combined effects of thermal radiation, thermophoresis, Brownian motion, magnetic field and variable viscosity on boundary layer flow, heat and mass transfer of an electrically conducting nanofluid on a radially stretching convectively heated sheet. The governing equations transformed into a system of ODEs by using suitable similarity variables and solved numerically with RK4 method. Akbar et al.^8^ studied the two-dimensional non-transient incompressible viscous nanofluid flow on a stretching/shrinking plate. The governing PDEs were transformed into a set of ODEs by similarity variables and solved numerically via shooting method. Ramzan et al.^9^ studied the non-transient incompressible MHD nanofluid flow due to an infinite swirling disk with constant angular velocity, and the various velocity slip conditions are considered either. The governing equations were transformed into a set of nonlinear ODEs and solved numerically via the RK4 method. Alshomrani and Gul^10^ studied the nanofluid flow of a liquid film in a porous medium on a stretching sheet via the presence of velocity slip and thermal slip. The governing equations were transformed into a set of ODEs via suitable similarity variables and solved via the Homotopy Analysis Method (HAM). Gul and Sohail^11^ investigated the various Marangoni convection over a thin film flow on a stretching cylinder. The suitable similarity variables transformed the governing equations of this study into a set of ODEs and solved numerically via RK4 method. Ellahi^12^ investigated the MHD non-Newtonian nanofluid flow inside a pipe with an assumption that temperature of the pipe was higher than fluid temperature and also considered two particular temperature dependent viscosity models. The governing equations were transformed into a set of ODEs via suitable similarity variables and solved by the HAM. The analytical solutions of velocity field, the temperature distribution, and nano concentration have been derived. Khan and Pop^13^ investigated the steady two-dimensional laminar nano fluid flow and heat transfer arising from the stretching of a sheet and Brownian motion and thermophoresis was also considered in the problem. The governing equations were solved numerically after transforming the governing PDEs into a set of ODEs. Mustafa et al.^14^ studied the incompressible nanofluid flow, heat and mass transfer in a channel with presence of Brownian motion and thermophoresis effects. The governing equations were converted from PDEs into ODEs using suitable similarity transformation and then solved with both numerical method of RK4 and analytically with HAM. Akbar and Nadeem^15^ studied the two dimensional incompressible steady peristaltic flow of a nanofluid flow, heat, and mass transfer in an endoscope. The governing equations were transformed into dimensionless form and solved analytically via the Homotopy Perturbation method (HPM). Lakshmisha et al.^16^ investigated the three-dimensional transient laminar motion of a viscous, incompressible MHD fluid flow and heat transfer caused by the stretching of an infinite flat surface. The fluid was stationary at infinity, and the no-slip condition was imposed at the stretching surface in two lateral directions, where suction or injection can be applied. The governing equations were reduced into ODEs and solved by three different numerical methods. Wang^17^ investigated three-dimensional fluid flow due to the stretching of a sheet in two directions. The governing equations were reduced into a set of ODEs via suitable similarity transformation and then solved by the numerical method of RK4. Ahmad et al.^18^ investigated the problem of forced convection boundary layer nanofluid flow and heat transfer from a stationary semi-infinite flattish sheet and another problem similar to the previous one, but this time the flat sheet was not stationary. The governing equations were converted into a set of ODEs by a transformation and then solved with the numerical method of RK4. Chamkha et al.^19^ investigated the problem of boundary-layer nanofluid flow, heat and mass transfer on a dynamic porous media in the presence of magnetic field, heat generation or absorption, thermophoresis, Brownian motion, and suction or injection effects. The governing equations were reduced into a system of ODEs and solved numerically via the finite difference method (FDM). Kandasamy et al.^20^ studied the problem of three-dimensional unsteady laminar nanofluid flow, heat, and mass transfer due to the stretchy perpendicular sheet with changing stream conditions in the presence of Brownian motion and thermophoresis effects. The governing equations were reduced into a system of coupled nonlinear ODEs and solved numerically with the Oberbeck–Boussinesq approximation. Berkan et al.^21^ studied the problem of intransient three-dimensional condensation film over an angled swirling disk. The governing equations were reduced into a set of ODEs via transformation and solved analytically with AGM. The results were compared with the previously published studies. Mirgolbabaee et al.^22^ studied a two-dimensional intransient MHD laminar flow of fluid along parallel porous walls in which fluid is uniformly injected or removed. The governing equations were reduced into a set of ODEs via a similarity transformation and solved analytically. Jalili et al.^23^ studied the impacts of angled Lorentz body force and changing viscosity for the flow of non-Newtonian Williamson nanofluid over a stretchy sheet. The governing equations were transformed into ODEs via similarity variables and solved analytically. Jalili et al.^24^ studied the flow of an intransient two-dimensional MHD nanofluid over a semi-infinite stretchy flat plate. The governing equations were reduced into a set of ODEs and solved analytically. Jalili et al.^25^ investigated the problem of two-dimensional steady boundary layer micropolar ferrofluid flow and heat transfer due to the constricting plate with presence of thermal radiation and transverse magnetic field. The governing equations reduced into system of ODEs and solved analytically and numerically. Jalili et al.^26^ proposed the Hybrid Analytical and Numerical method (the HAN method) for solving a the problem of viscous, incompressible, laminar axisymmetric flow of a micropolar fluid with presence of magnetic field between two stretchable disks. The governing equations were reduced into ODEs by similarity variables and solved analytically. Jalili et al.^27,28^ used the same method of HAN in two other studied either. Many problems^29–36^ related to fluid mechanics were studied and used the similarity transformation to convert the PDEs into ODEs but they solved numerically, Meanwhile, the modified HAN or AGM method had the potential to solve these problems analytically. The novelty of this article is that these two methods were used and the analytical answer was obtained.

This paper investigates heat and mass transfer in a transient swirling angled sheet analytically with two techniques for a 3D thin nanomaterial film flow. The semi-analytical methods used in this study are modified AGM and HAN methods. The modified AGM is novel and has made the former AGM more complete. The second semi-analytical technique is the HAN method, which is applied because Zeeshan et al. already solved the numerical solution to this problem^37^. The results from these two semi-analytical solutions were compared with the previously published RK4 solution.

## Mathematical description

**Figure 1:**
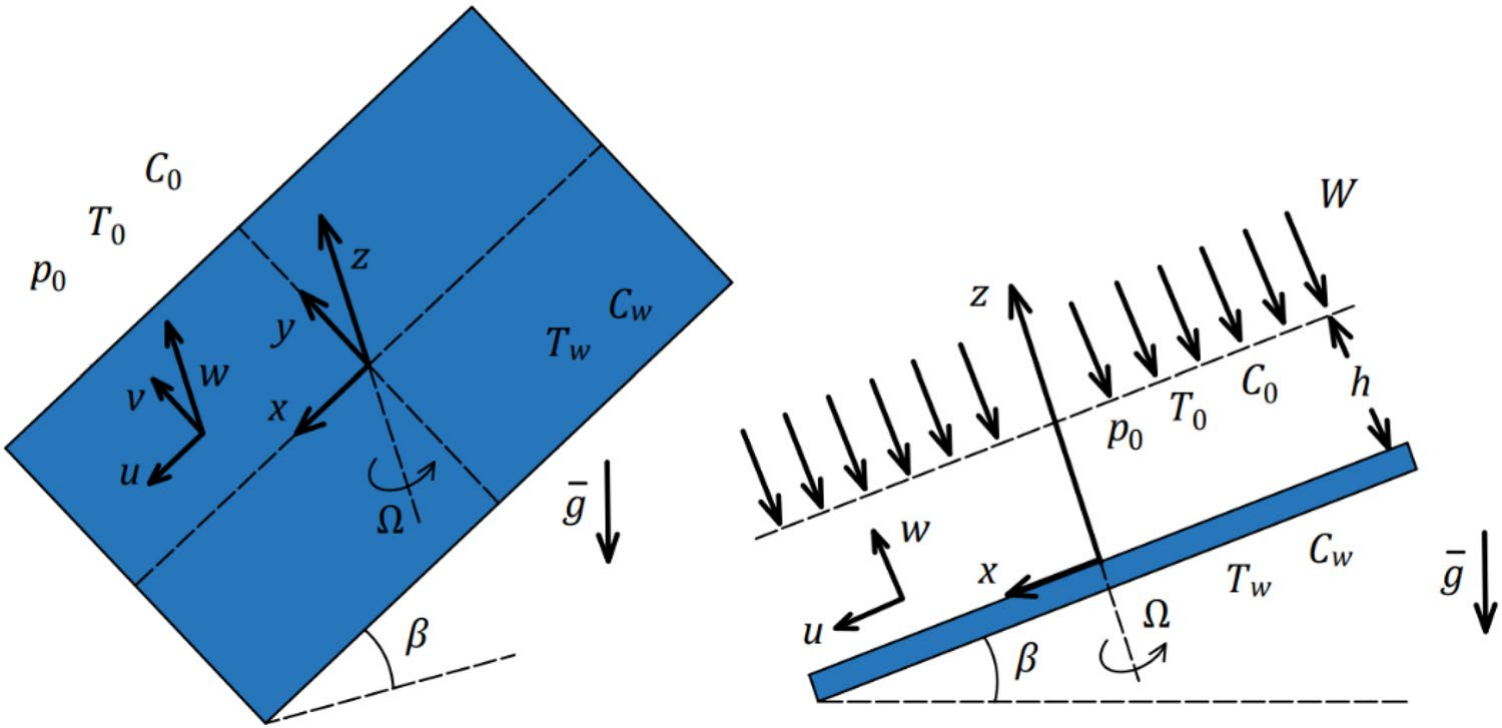
The geometry of the problem.

The geometry of the study is a swirling sheet with a three-dimensional transient nanomaterial thin-film moment, as illustrated in Fig. 1. The plate swirls with the angular velocity of $\Omega ,$  and the inclined plate has an angle of $\beta$ with the horizon. The nanomaterial thickness of the sheet is indicated by $h$, and the speed of the sprayed fluid is denoted by $W$. The terminal effect is neglected because the thickness of the fluid film in comparison to the radius of the disc is not thick enough. The gravitation force exists, and it is denoted by $\overline{g}$ , and its direction is illustrated in the following figure. The temperature of the film surface is denoted by ${T}_{0}$. The temperature of the inclined swirling surface is denoted by ${T}_{w}$. The concentration of the film surface is denoted by ${C}_{0}$. The concentration of the inclined swirling surface is denoted by ${C}_{w}$.

The thickness of the fluid film is very thin, and the pressure at the surface of the surface is denoted by ${p}_{0}$ and it is just a function of $z$. The viscous dissipation function in the energy equation is negligible. The governing equations of the problem are as follows^2,3,5,6,8,37^:

The equation of conservation of mass:

1 $$\nabla \cdot \mathbf{v}=0 .$$

The equation of conservation of momentum in $x$ direction:

2 $${\rho }_{f}\frac{Du}{Dt}=\mu {\nabla }^{2}u+{\rho }_{f}\overline{g}\mathrm{sin }\left(\beta \right) .$$

The equation of conservation of momentum in $y$ direction:

3 $${\rho }_{f}\frac{Dv}{Dt}=\mu {\nabla }^{2}v .$$

The equation of conservation of momentum in $z$ direction:

4 $${\rho }_{f}\frac{Dw}{Dt}=\mu {\nabla }^{2}w-{\rho }_{f}\overline{g}\mathrm{cos }\left(\beta \right)-{p}_{z} .$$

The equation of conservation of energy:

5 $$\frac{DT}{Dt}=\alpha {\nabla }^{2}T-\frac{{\left(\rho {c}_{p}\right)}_{p}}{{\left(\rho {c}_{p}\right)}_{f}}\left[{D}_{B}\nabla C\cdot \nabla T+\frac{{D}_{T}}{T}\nabla T\cdot \nabla T\right]$$

The equation of conservation of concentration:

6 $$\frac{DC}{Dt}={D}_{B}{\nabla }^{2}C+\frac{{D}_{T}}{{T}_{h}}{\nabla }^{2}T ,$$

where $D/Dt$ denotes the total derivative to the variable of time, $\nabla$ is the gradient operator, $u$, $v,$ and $z$ are the velocities in the $x$, $y,$ and $z$ directions, respectively, ${\nabla }^{2}$ is the Laplacian operator, $T$ is the temperature, $C$ is the concentration, ${\rho }_{f}$, is the density of the base fluid, $\mu$ is the dynamic viscosity, $\alpha$ is the thermal diffusivity, ${c}_{p}$, is the specific heat capacity at a constant pressure of nanofluid, ${\left(\rho {c}_{p}\right)}_{p}/{\left(\rho {c}_{p}\right)}_{f}$ , is the ratio of nanoparticles' heat capacity to the base fluid heat capacity, ${D}_{B}$ is the Brownian diffusion coefficient, and ${D}_{T}$, is the thermophoretic diffusion coefficient.

The corresponding boundary conditions of Eqs. (1)–(6) are as follows:

7 $$\begin{aligned}&u=-\Omega y, v=-\Omega x, w=0,T={T}_{w}, C={C}_{w},\quad \mathrm{at} z=0\\ &u=0, v=0, w=-W,p={p}_{0}, T={T}_{0}, C={C}_{0},\quad \mathrm{at} z=h\end{aligned}$$

The similarity transformations are considered as follows^8,11,37^:

8 $$\begin{aligned}u&=\frac{-\Omega y}{\sqrt{1-bt}}g\left(\xi \right)+\frac{\Omega x}{\sqrt{1-bt}}{f}^{\prime}\left(\xi \right)+\frac{\overline{g} }{\sqrt{1-bt}}k\left(\xi \right)\mathrm{sin}\left(\frac{\beta }{{\Omega }^{\prime}}\right) ,\\ v&=\frac{-\Omega x}{\sqrt{1-bt}}g\left(\xi \right)+\frac{\Omega y}{\sqrt{1-bt}}{f}^{\prime}\left(\xi \right)+\frac{\overline{g} }{\sqrt{1-bt}}h\left(\xi \right)\mathrm{sin}\left(\frac{\beta }{{\Omega }^{\prime}}\right) ,\\ w&=-2\sqrt{\frac{\Omega \nu }{1-bt}}f\left(\xi \right) , \theta =\frac{T-{T}_{w}}{{T}_{h}-{T}_{w}} , \phi =\frac{C-{C}_{w}}{{C}_{h}-{C}_{w}} ,\xi =z\sqrt{\frac{\Omega }{\nu \left(1-bt\right)}} ,\end{aligned}$$

Here, $\nu$ is the kinematic viscosity, $\theta$ is the dimensionless temperature, and $\phi$ is the dimensionless concentration. The similarity variables of Eq. (8) can be substituted in Eqs. (2)–(6) for converting a system of nonlinear PDEs into a system of nonlinear dimensionless coupled ODEs:

9 $${f}^{^{\prime\prime\prime}}-{\left({f}^{\prime}\right)}^{2}+{\mathrm{g}}^{2}+2f{f}^{^{\prime\prime}}-S\left({f}^{\prime}+\frac{\xi }{2}{f}^{^{\prime\prime}}\right)=0,$$

10 $${\mathrm{g}}^{^{\prime\prime}}-2\mathrm{g}{f}^{\prime}+2f{\mathrm{g}}^{{\prime}}-S\left(\mathrm{g}+\frac{\xi }{2}{\mathrm{g}}^{{\prime}}\right)=0,$$

11 $${k}^{^{\prime\prime}}-k{f}^{\prime}+h\mathrm{g}+2f{k}^{\prime}+1-\frac{S}{2}\left(k+\xi {k}^{\prime}\right)=0,$$

12 $${h}^{^{\prime\prime}}-k\mathrm{g}-h{f}^{\prime}+2fh^{\prime}-\frac{S}{2}\left(h-\xi {h}^{\prime}\right)=0,$$

13 $${\theta }^{^{\prime\prime}}+2Prf{\theta }^{\prime}+Nb{\phi }^{\prime}{\theta }^{\prime}+Nt{\left(\theta ^{\prime}\right)}^{2}+\frac{S}{2}\left(\xi {\theta }^{\prime}+{\xi }^{2}\theta ^{\prime\prime}\right)=0,$$

14 $${\phi }^{^{\prime\prime}}+2Scf{\phi }^{\prime}+\left(\frac{Nt}{Nb}\right){\theta }^{^{\prime\prime}}+\frac{S}{2}\left(\xi {\phi }^{\prime}+{\xi }^{2}\phi ^{\prime\prime}\right)=0.$$

Substituting the similarity variables of Eq. (8) in Eq. (7) will be as follows:

15 $$\begin{aligned}&f=0, {f}^{\prime}=0, \mathrm{g}=1,k=0, h=0,\uptheta =0,\upphi =0, \mathrm{at} \xi =0\\& f^{\prime\prime}=0,\mathrm{g^{\prime}}=0,k^{\prime}=0, h^{\prime}=0,\uptheta =1,\upphi =1, \mathrm{at} \xi =\delta\end{aligned}$$

where $Pr$ is the Prandtl number, $Sc$ is the Schmidt number, $Nb$ is the Brownian motion parameter, $S$ is the parameter that depends on the angular velocity of the rotating surface, and $Nt$ is the thermophoretic parameter, which is defined as^37,38^:

16 $$\begin{aligned}&Pr=\nu /\alpha,\,Sc=\mu /{D}_{B},\,Nb=\left[{\left(\rho c\right)}_{p}{D}_{B}\left({C}_{w}-{C}_{h}\right)\right]/\left[{\left(\rho c\right)}_{f}\nu \right],\\& Nt=\left[{\left(\rho c\right)}_{p}{D}_{T}\left({T}_{w}-{T}_{h}\right)\right]/\left[{\left(\rho c\right)}_{f}\nu {T}_{h}\right],\, S=1/\Omega \end{aligned}$$

The constant normalized thickness of $\delta$ is as follows^37^:

17 $$\delta =\varepsilon \sqrt{\frac{\Omega }{\nu \left(1-bt\right)}} , (17)$$

The dimensionless Nusselt and Sherwood numbers are as follows^37^:

18 $$Nu=\frac{{\left(\frac{\partial T}{\partial z}\right)}_{w}}{{T}_{h}-{T}_{w}}=\delta {\theta }^{\prime}\left(0\right)$$

19 $$Sh=\frac{{\left(\frac{\partial C}{\partial z}\right)}_{w}}{{C}_{h}-{C}_{w}}=\delta {\phi }^{\prime}\left(0\right)$$

### Methodology

### Description of the HAN method

Jalili et al.^26–28^ developed the HAN method for approximating an analytical solution for a differential equation. In this part, the explanation of the HAN method is as follows:

Consider an ODE of the m $th$ order as follow:

20 $$\Gamma \left(\zeta \left(\upxi \right),{\zeta }^{\prime}\left(\upxi \right),{\zeta }^{{\prime\prime}}\left(\upxi \right),\ldots , {\zeta }^{\left(m\right)}\left(\upxi \right)\right)=0 .$$

Equation (20) is a nonlinear differential equation, and $\Gamma$ is the function of $\zeta$ and its derivatives to $\upxi$. The parameter $\zeta$ is the function of the independent variable $\upxi$. The derivatives of the function $\zeta \left(\upxi \right)$ with respect to $\upxi$ at $\upxi =0$ and $\upxi =L$ are denoted as follows:

21 $$\left\{\begin{array}{l}\zeta \left(\upxi \right)={\zeta }_{0} , {\zeta }^{\prime}\left(\upxi \right)={\zeta }_{1} ,\ldots {,\zeta }^{\left(m-1\right)}\left(\upxi \right)={\zeta }_{m-1}\quad \text{when } \xi =0,\\ \zeta \left(\upxi \right)={\zeta }_{{L}_{0}} , {\zeta }^{\prime}\left(\upxi \right)={\zeta }_{{L}_{1}} ,\ldots {,\zeta }^{\left(m-1\right)}\left(\upxi \right)={\zeta }_{{L}_{m-1}} \quad \text{when }\xi =L .\end{array}\right.$$

The solution of Eq. (20) is considered as follows:

22 $$\zeta \left(\upxi \right)=\sum_{i=0}^{n}{a}_{i}{\upxi }^{i}={a}_{0}+{a}_{1}{\upxi }^{1}+{a}_{2}{\upxi }^{2}+\cdots +{a}_{n}{\upxi }^{n},$$

Here, ${a}_{0}$, ${a}_{1}$, …, ${a}_{n}$ are $n+1$ constant coefficients which $n>m$. By solving a system of $n+1$ unknowns and $n+1$ equations, constant coefficients will be determined. The boundary conditions of the problem can construct some of these equations as follows:

23 $$\left\{\begin{array}{l}\zeta \left(0\right)={a}_{0}={\zeta }_{0} ,\\ {\zeta }^{\prime}\left(0\right)={a}_{1}={\zeta }_{1 },\\ {\zeta }^{^{\prime\prime}}\left(0\right)={a}_{2}={\zeta }_{2} ,\\ \cdots\\ \cdots\\ \end{array}\right.$$

24 $$\left\{\begin{array}{l}\zeta \left(L\right)={a}_{0}+{a}_{1}L+{a}_{2}{L}^{2}+\cdots +{a}_{n}{L}^{n}={\zeta }_{{L}_{0}} , \\ {\zeta }^{\prime}\left(L\right)={a}_{1}+2{a}_{2}L+{3a}_{3}{L}^{2}+\cdots +{na}_{n}{L}^{n-1}={\zeta }_{{L}_{1}} , \\ {\zeta }^{^{\prime\prime}}\left(L\right)={2a}_{2}+6{a}_{3}L+{12a}_{4}{L}^{2}+\cdots +{n\left(n-1\right)a}_{n}{L}^{n-2}={\zeta }_{{L}_{2}} , \\ \cdots\\ \cdots\\ \end{array}\right.$$

The constructed equations from boundary conditions of the problem as they can be seen in Eqs. (23), (24) are limited because we assume the value of $n$ is higher than $m$ earlier in this methodology. But more boundary equations are needed, and a numerical method (no matter which numerical method and no matter what kind of software package) can approximate these additional boundary conditions for making the remaining needed equations. So, the new approximated boundary conditions are as follows:

25 $$\left\{\begin{array}{l}\zeta \left(\upxi \right)={\alpha }_{0} , {\zeta }^{\prime}\left(\upxi \right)={\alpha }_{1} ,\ldots {,\zeta }^{\left(m-1\right)}\left(\upxi \right)={\alpha }_{m-1} at \xi ={L}_{0} ,\\ \zeta \left(\upxi \right)={\beta }_{0} , {\zeta }^{\prime}\left(\upxi \right)={\beta }_{1} ,\ldots {,\zeta }^{\left(m-1\right)}\left(\upxi \right)={\beta }_{m-1} at \xi ={L}_{1} ,\\ \zeta \left(\upxi \right)={\gamma }_{0} , {\zeta }^{\prime}\left(\upxi \right)={\gamma }_{1} ,\ldots {,\zeta }^{\left(m-1\right)}\left(\upxi \right)={\gamma }_{m-1} at \xi ={L}_{2} ,\\ \cdots\\ \cdots\\ \zeta \left(\upxi \right)={\varepsilon }_{0} , {\zeta }^{\prime}\left(\upxi \right)={\varepsilon }_{1} ,\ldots {,\zeta }^{\left(m-1\right)}\left(\upxi \right)={\varepsilon }_{m-1} at \xi ={L}_{z} .\end{array}\right.$$

For instance, the following equations are constructed from approximated boundary conditions of Eq. (25):

26 $$\left\{\begin{array}{l}\zeta \left({L}_{0}\right)={a}_{0}+{a}_{1}\left({L}_{0}\right)+{a}_{2}{\left({L}_{0}\right)}^{2}+\cdots +{a}_{n}{\left({L}_{0}\right)}^{n}={\alpha }_{0} , \\ {\zeta }^{\prime}\left({L}_{0}\right)={a}_{1}+2{a}_{2}\left({L}_{0}\right)+{3a}_{3}{\left({L}_{0}\right)}^{2}+\cdots +{na}_{n}{\left({L}_{0}\right)}^{n-1}={\alpha }_{1} , \\ {\zeta }^{^{\prime\prime}}\left({L}_{0}\right)={2a}_{2}+6{a}_{3}\left({L}_{0}\right)+{12a}_{4}{\left({L}_{0}\right)}^{2}+\cdots +{n\left(n-1\right)a}_{n}{\left({L}_{0}\right)}^{n-2}={\alpha }_{2 ,} \\ \cdots\\\cdots\\ {\left({\zeta }^{\left(m-1\right)}\left(\upxi \right)\right)}_{\upxi ={L}_{0}}={\alpha }_{m-1} .\\ \end{array} \right.$$

27 $$\left\{\begin{array}{l}\zeta \left({L}_{1}\right)={a}_{0}+{a}_{1}\left({L}_{1}\right)+{a}_{2}{\left({L}_{1}\right)}^{2}+\cdots +{a}_{n}{\left({L}_{1}\right)}^{n}={\beta }_{0} , \\ {\zeta }^{\prime}\left({L}_{1}\right)={a}_{1}+2{a}_{2}\left({L}_{1}\right)+{3a}_{3}{\left({L}_{1}\right)}^{2}+\cdots +{na}_{n}{\left({L}_{1}\right)}^{n-1}={\beta }_{1} , \\ {\zeta }^{^{\prime\prime}}\left({L}_{1}\right)={2a}_{2}+6{a}_{3}\left({L}_{1}\right)+{12a}_{4}{\left({L}_{1}\right)}^{2}+\cdots +{n\left(n-1\right)a}_{n}{\left({L}_{1}\right)}^{n-2}={\beta }_{2} ,\\ \cdots\\ \cdots\\ {\left({\zeta }^{\left(m-1\right)}\left(\upxi \right)\right)}_{\upxi ={L}_{1}}={\beta }_{m-1} .\\ \end{array} \right.$$

28 $$\left\{\begin{array}{l}\zeta \left({L}_{2}\right)={a}_{0}+{a}_{1}\left({L}_{2}\right)+{a}_{2}{\left({L}_{2}\right)}^{2}+\cdots +{a}_{n}{\left({L}_{2}\right)}^{n}={\gamma }_{0} , \\ {\zeta }^{\prime}\left({L}_{2}\right)={a}_{1}+2{a}_{2}\left({L}_{2}\right)+{3a}_{3}{\left({L}_{2}\right)}^{2}+\cdots +{na}_{n}{\left({L}_{2}\right)}^{n-1}={\gamma }_{1} , \\ {\zeta }^{^{\prime\prime}}\left({L}_{2}\right)={2a}_{2}+6{a}_{3}\left({L}_{2}\right)+{12a}_{4}{\left({L}_{2}\right)}^{2}+\cdots +{n\left(n-1\right)a}_{n}{\left({L}_{2}\right)}^{n-2}={\gamma }_{2} ,\\ \cdots\\ \cdots\\ {\left({\zeta }^{\left(m-1\right)}\left(\upxi \right)\right)}_{\upxi ={L}_{2}}={\gamma }_{m-1} .\\ \end{array} \right.$$

29 $$\left\{\begin{array}{l}\zeta \left({L}_{z}\right)={a}_{0}+{a}_{1}\left({L}_{z}\right)+{a}_{2}{\left({L}_{z}\right)}^{2}+\cdots +{a}_{n}{\left({L}_{z}\right)}^{n}={\varepsilon }_{0} , \\ {\zeta }^{\prime}\left({L}_{z}\right)={a}_{1}+2{a}_{2}\left({L}_{z}\right)+{3a}_{3}{\left({L}_{z}\right)}^{2}+\cdots +{na}_{n}{\left({L}_{z}\right)}^{n-1}={\varepsilon }_{1} , \\ {\zeta }^{^{\prime\prime}}\left({L}_{z}\right)={2a}_{2}+6{a}_{3}\left({L}_{z}\right)+{12a}_{4}{\left({L}_{z}\right)}^{2}+\cdots +{n\left(n-1\right)a}_{n}{\left({L}_{z}\right)}^{n-2}={\varepsilon }_{2}, \\ \cdots\\ \cdots\\ {\left({\zeta }^{\left(m-1\right)}\left(\upxi \right)\right)}_{\upxi ={L}_{z}}={\varepsilon }_{m-1} .\\ \end{array} \right.$$

**Figure 2:**
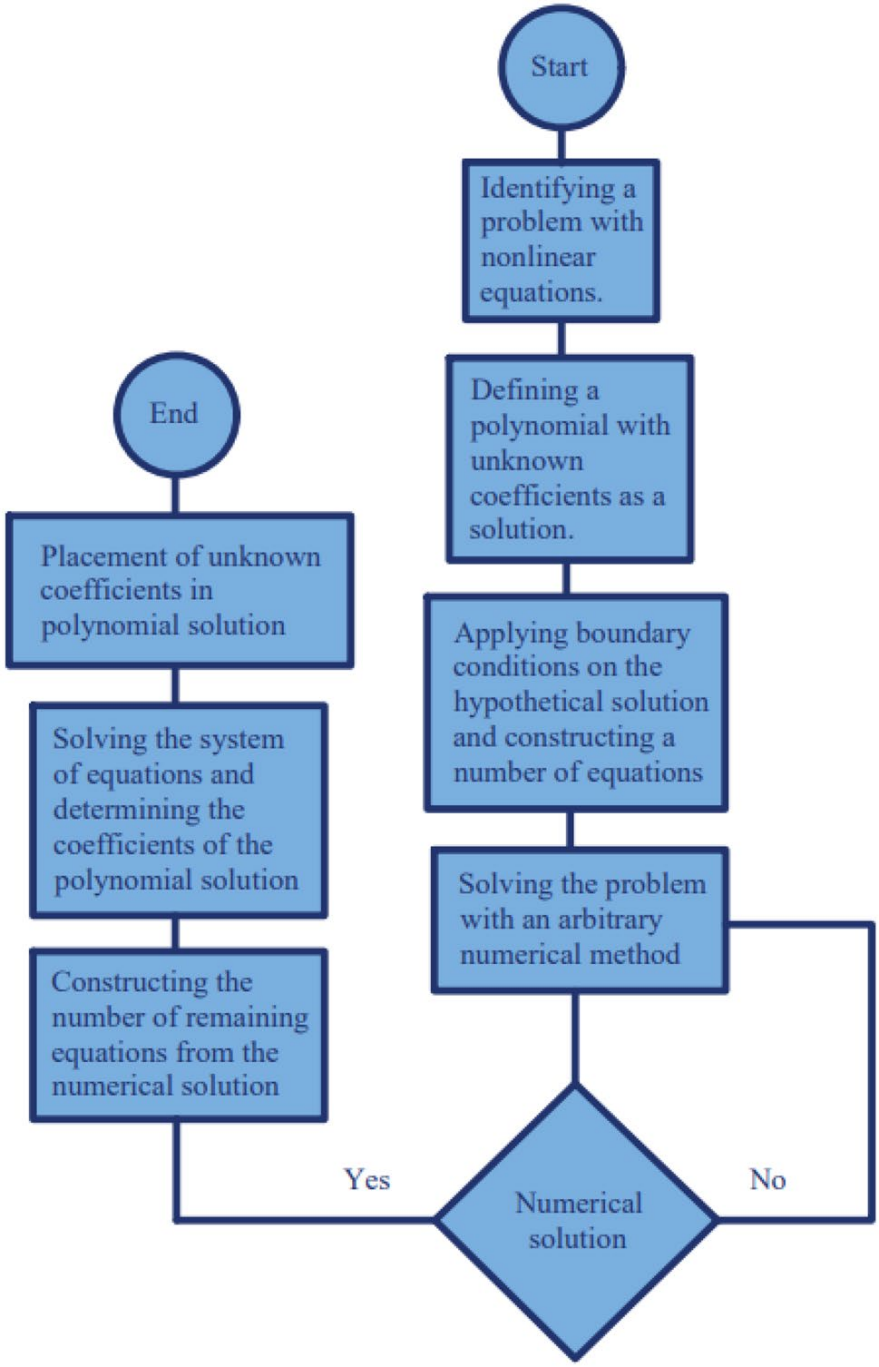
The flow chart of the HAN method.

According to Eqs. (26)–(29), it can be derived as many equations as possible are needed to create a system with $n+1$ equations and $n+1$ unknowns. The limitation of the HAN method is just in the numerical method that is used, and this means that if no numerical method could solve a problem, the HAN method could not be used because this method seriously needs a numerical solution. To summarize the mentioned method in a more compact form, the following Fig. 2, the flow chart is presented for the HAN method:

### Application of the HAN method

For applying the HAN method, let us assume the following functions are the semi-analytical solutions of Eqs. (9)–(14):

30 $$\begin{aligned}&f\left(\upxi \right)=\sum_{i=0}^{7}{a}_{i}{\upxi }^{i} ,\quad \mathrm{g}\left(\upxi \right)=\sum_{i=0}^{6}{b}_{i}{\upxi }^{i} ,\quad k\left(\upxi \right)=\sum_{i=0}^{6}{c}_{i}{\upxi }^{i} ,\\ & h\left(\upxi \right)=\sum_{i=0}^{6}{d}_{i}{\upxi }^{i},\quad\uptheta \left(\upxi \right)=\sum_{i=0}^{6}{e}_{i}{\upxi }^{i} ,\quad\upphi \left(\upxi \right)=\sum_{i=0}^{6}{w}_{i}{\upxi }^{i} ,\end{aligned}$$

Based on Eq. (30), there are 43 unknown coefficients, and 43 equations are needed to obtain them. Equation (15) makes only 13 equations, and the remaining 30 must be made numerically. This study used the numerical solution of Zeeshan et al.^37^. Finally, according to Table 1, the system of ODEs of Eqs. (9)–(14) for 4 cases can be solved by calculating the system of 46 equations and 46 unknowns and the solutions of Eqs. (9)–(14) for all available cases in Table 1 are as follows:

**Table 1:** Different cases of the study.

Case number	$Pr$	$Nt$	$Nb$	$Sc$	$S$	$\delta$
1	6.6	0.2	0.2	2.0	0.0	1.0
2	6.7	0.4	0.4	4.0	0.3	1.0
3	7.1	0.6	0.6	6.0	0.5	1.0
4	7.3	0.8	0.8	8.0	0.6	1.0

Solutions of case 1 where $Pr=6.6$, $Nt=0.2$, $Nb=0.2$, $Sc=2.0$, $S=0.0$, $\delta =1.0$ are are demostrated in Eqs. (31)–(36) as follows:

31 $$\begin{aligned}f\left(\upxi \right)& =0.0002732904460 {\upxi }^{7} - 0.001130398705 {\upxi }^{6} - 0.002983457929 {\upxi }^{5} \\ &\quad + 0.03142026994 {\upxi }^{4}- 0.1667247673 {\upxi }^{3} + 0.3527041428 {\upxi }^{2}\end{aligned}$$

32 $$\begin{aligned}\mathrm{g}\left(\upxi \right)&=-0.001545033804 {\upxi }^{6}+ 0.01589724438 {\upxi }^{5}- 0.09880567227 {\upxi }^{4} \\ &\quad + 0.2325237815 {\upxi }^{3}+ 0.0005790233777 {\upxi }^{2}- 0.3737227213\upxi + 1\end{aligned}$$

33 $$\begin{aligned}k(\upxi )&=-0.002279700115 {\upxi }^{6}+ 0.01147406663 {\upxi }^{5}- 0.01381462277 {\upxi }^{4} \\ &\quad + 0.04064226334 {\upxi }^{3}- 0.5005751938 {\upxi }^{2}+ 0.8907899562\upxi \end{aligned}$$

34 $$\begin{aligned}h\left(\upxi \right)&=-0.001567686924 {\upxi }^{6}+ 0.01446119756 {\upxi }^{5}- 0.07067881448 {\upxi }^{4} \\ &\quad + 0.1490380770 {\upxi }^{3}- 0.0001265692365 {\upxi }^{2}- 0.2270457008\upxi \end{aligned}$$

35 $$\begin{aligned}\uptheta \left(\upxi \right)&=-0.2116669756 {\upxi }^{6}+ 0.8465834245 {\upxi }^{5}- 1.029402144 {\upxi }^{4} \\ &\quad + 0.2315199252 {\upxi }^{3}- 0.3564099429 {\upxi }^{2}+ 1.519375713\upxi \end{aligned}$$

36 $$\begin{aligned}\upphi \left(\upxi \right)&=0.2829625151 {\upxi }^{6}- 1.039481420 {\upxi }^{5}+ 1.103469812 {\upxi }^{4} \\ &\quad - 0.2898952916 {\upxi }^{3}+ 0.3681095391 {\upxi }^{2}+ 0.5748348455\upxi \end{aligned}$$

Solutions of case 2 where $Pr=6.7$, $Nt=0.4$, $Nb=0.4$, $Sc=4.0$, $S=0.3$, $\delta =1.0$ are are demostrated in Eqs. (37)–(42) as follows:

37 $$\begin{aligned}f\left(\upxi \right)&=-0.0002079022321 {\upxi }^{7}+ 0.001973475990 {\upxi }^{6} \\ &\quad - 0.01470749656 {\upxi }^{5}+ 0.05432728425 {\upxi }^{4}- 0.1666873768 {\upxi }^{3}+ 0.2959371975 {\upxi }^{2} \end{aligned}$$

38 $$\begin{aligned}\mathrm{g}\left(\upxi \right)&=-0.005133576574 {\upxi }^{6}+ 0.02908484722 {\upxi }^{5} \\ &\quad - 0.09024744620 {\upxi }^{4}+ 0.1540695922 {\upxi }^{3}+ 0.1508920312 {\upxi }^{2}- 0.5176258308\upxi + 1 \end{aligned}$$

39 $$\begin{aligned}k\left(\upxi \right)&=-0.001496251208 {\upxi }^{6}+ 0.01038296954 {\upxi }^{5} \\ &\quad - 0.03189600552 {\upxi }^{4}+ 0.07790099004 {\upxi }^{3}- 0.5003734521 {\upxi }^{2}+ 0.8516906158\upxi \end{aligned}$$

40 $$\begin{aligned}h\left(\upxi \right)&=-0.002786510359 {\upxi }^{6}+ 0.02018172983 {\upxi }^{5} \\ &\quad - 0.07691644778 {\upxi }^{4}+ 0.1414225437 {\upxi }^{3}+ 0.00009716095831 {\upxi }^{2}- 0.2009857489\upxi \end{aligned}$$

41 $$\begin{aligned}\uptheta \left(\upxi \right)&=-0.1359384154 {\upxi }^{6}+ 0.4518613532 {\upxi }^{5} \\ &\quad - 0.4083916572 {\upxi }^{4}+ 0.08938493236 {\upxi }^{3}- 0.7445633333 {\upxi }^{2}+ 1.747647120\upxi \end{aligned}$$

42 $$\begin{aligned}\upphi \left(\upxi \right)&=0.1778020637 {\upxi }^{6}- 0.4076008170 {\upxi }^{5} \\ &\quad + 0.03931969082 {\upxi }^{4}+ 0.03147310942 {\upxi }^{3}+ 0.7127832489 {\upxi }^{2}+ 0.4462227042\upxi \end{aligned}$$

Solutions of case 3 where $Pr=7.1$, $Nt=0.6$, $Nb=0.6$, $Sc=6.0$, $S=0.5$, $\delta =1.0$ are are demostrated in Eqs. (43)–(48) as follows:

43 $$\begin{aligned}f\left(\upxi \right)&=-0.0006778087691 {\upxi }^{7}+ 0.004625490183 {\upxi }^{6}- 0.02250363290 {\upxi }^{5}\\ &\quad + 0.06737914699 {\upxi }^{4}- 0.1666335480 {\upxi }^{3}+ 0.2655137223 {\upxi }^{2} \end{aligned}$$

44 $$\begin{aligned}\mathrm{g}\left(\upxi \right)&=-0.006002419664 {\upxi }^{6}+ 0.03178524881 {\upxi }^{5}- 0.07449152309 {\upxi }^{4}\\ &\quad + 0.09470427126 {\upxi }^{3}+ 0.2511832131 {\upxi }^{2}- 0.6114248738\upxi + 1 \end{aligned}$$

45 $$\begin{aligned}k\left(\upxi \right)&=-0.001619303915 {\upxi }^{6}+ 0.01124629570 {\upxi }^{5}- 0.04380530174 {\upxi }^{4}\\ &\quad + 0.1009044393 {\upxi }^{3}- 0.5002496563 {\upxi }^{2}+ 0.8264915466\upxi \end{aligned}$$

46 $$\begin{aligned}h\left(\upxi \right)&=-0.003463347867 {\upxi }^{6}+ 0.02342157653 {\upxi }^{5}- 0.08036919805 {\upxi }^{4}\\ &\quad + 0.1364751684 {\upxi }^{3}+ 0.0002471443773 {\upxi }^{2}- 0.1847707971\upxi \end{aligned}$$

47 $$\begin{aligned}\uptheta \left(\upxi \right)&=-0.05306295007 {\upxi }^{6}+ 0.1129913194 {\upxi }^{5}\\ &\quad - 0.01888402561 {\upxi }^{4}+ 0.2453130864 {\upxi }^{3}- 1.328564902 {\upxi }^{2}+ 2.042207471\upxi \end{aligned}$$

48 $$\begin{aligned}\upphi \left(\upxi \right)&=-0.01401617829 {\upxi }^{6}+ 0.3947414707 {\upxi }^{5}\\ &\quad - 0.9858640302 {\upxi }^{4}+ 0.1284080241 {\upxi }^{3}+ 1.244433830 {\upxi }^{2}+ 0.2322968833\upxi \end{aligned}$$

Solutions of case 4 where $Pr=7.3$, $Nt=0.8$, $Nb=0.8$, $Sc=8.0$, $S=0.6$, $\delta =1.0$ are are demostrated in Eqs. (49)–(54) as follows:

49 $$\begin{aligned}f\left(\upxi \right)&=-0.0009410950258 {\upxi }^{7}+ 0.006062772127 {\upxi }^{6}\\ &\quad - 0.02636138926 {\upxi }^{5}+ 0.07333935113 {\upxi }^{4}- 0.1665967652 {\upxi }^{3}+ 0.2521894950 {\upxi }^{2}\end{aligned}$$

50 $$\begin{aligned}\mathrm{g}\left(\upxi \right)&=-0.006015080732 {\upxi }^{6}+ 0.03148047968 {\upxi }^{5}\\ &\quad - 0.06385582722 {\upxi }^{4}+ 0.06290813817 {\upxi }^{3}+ 0.3013300935 {\upxi }^{2}- 0.6572732066\upxi + 1\end{aligned}$$

51 $$\begin{aligned}k\left(\upxi \right)&=-0.001840200974 {\upxi }^{6}+ 0.01208551550 {\upxi }^{5}\\ &\quad - 0.04972369728 {\upxi }^{4}+ 0.1119001658 {\upxi }^{3}- 0.5001929272 {\upxi }^{2}+ 0.8141937745\upxi\end{aligned}$$

52 $$\begin{aligned}h\left(\upxi \right)&=-0.003764560601 {\upxi }^{6}+ 0.02487394812 {\upxi }^{5}\\ &\quad - 0.08187181977 {\upxi }^{4}+ 0.1340475806 {\upxi }^{3}+ 0.0003224592621 {\upxi }^{2}- 0.1770827583\upxi \end{aligned}$$

53 $$\begin{aligned}\uptheta \left(\upxi \right)&=0.02069658406 {\upxi }^{6}- 0.1078814027 {\upxi }^{5}\\ &\quad + 0.01694159650 {\upxi }^{4}+ 0.8363701696 {\upxi }^{3}- 2.151877171 {\upxi }^{2}+ 2.385750224\upxi \end{aligned}$$

54 $$\begin{aligned}\upphi \left(\upxi \right)&=-0.2282116667 {\upxi }^{6}+ 1.165647340 {\upxi }^{5}\\ &\quad - 1.727899009 {\upxi }^{4}- 0.1921012070 {\upxi }^{3}+ 2.017808929 {\upxi }^{2}- 0.03524438637\upxi \end{aligned}$$

### Description of the modified Akbari–Ganji method

The Akbari–Ganji Method was developed for solving nonlinear differential equations analytically. This method has solved many problems^21–25,39,40^ for which no exact analytical method exists. This paper introduces the modification of this method due to needing more accurate solutions.

To explain the main idea of modified AGM, the general form of the m $th$ order differential equation is assumed as:

55 $$\Theta :\Gamma \left(\zeta ,{\zeta }^{\prime},{\zeta }^{^{\prime\prime}},\ldots ,{\zeta }^{\left(m\right)}\right)=0; \zeta =\zeta \left(\upxi \right).$$

With boundary conditions:

56 $$\left\{\begin{array}{ll}\zeta \left(\upxi \right)={\zeta }_{0}, {\zeta }^{\prime}\left(\upxi \right)={\zeta }_{1},\ldots ,{\zeta }^{\left(m-1\right)}\left(\upxi \right)={\zeta }_{m-1} , &\quad at\ \xi =0\\ \zeta \left(\upxi \right)={\zeta }_{{L}_{0}}, {\zeta }^{\prime}\left(\upxi \right)={\zeta }_{{L}_{1}},\ldots ,{\zeta }^{\left(m-1\right)}\left(\upxi \right)={\zeta }_{{L}_{m-1}} ,&\quad at\ \xi =L\end{array}\right.$$

To solve Eq. (55), we can consider the answer as the following polynomial of degree $n$ with unknown constant coefficients:

57 $$\zeta \left(\upxi \right)=\sum_{i=0}^{n}{a}_{i}{\upxi }^{i}={a}_{0}+{a}_{1}{\upxi }^{1}+{a}_{2}{\upxi }^{2}+\cdots +{a}_{n}{\upxi }^{n},$$

Here, ${a}_{0}$, ${a}_{1}$, …, ${a}_{n}$ are $n+1$ constant coefficients which $n>m$. By solving a system of $n+1$ unknowns and $n+1$ equations, constant coefficients will be determined. The boundary conditions of the problem can construct some of these equations as follows:

58 $$\left\{\begin{array}{c}\zeta \left(0\right)={a}_{0}={\zeta }_{0} ,\\ {\zeta }^{\prime}\left(0\right)={a}_{1}={\zeta }_{1 },\\ {\zeta }^{^{\prime\prime}}\left(0\right)={a}_{2}={\zeta }_{2} ,\\ \cdots\\ \cdots\\ \end{array}\right.$$

59 $$\left\{\begin{array}{c}\zeta \left(L\right)={a}_{0}+{a}_{1}L+{a}_{2}{L}^{2}+\cdots +{a}_{n}{L}^{n}={\zeta }_{{L}_{0}} , \\ {\zeta }^{\prime}\left(L\right)={a}_{1}+2{a}_{2}L+{3a}_{3}{L}^{2}+\cdots +{na}_{n}{L}^{n-1}={\zeta }_{{L}_{1}} , \\ {\zeta }^{^{\prime\prime}}\left(L\right)={2a}_{2}+6{a}_{3}L+{12a}_{4}{L}^{2}+\cdots +{n\left(n-1\right)a}_{n}{L}^{n-2}={\zeta }_{{L}_{2}} , \\ \cdots\\ \cdots\\ \end{array}\right.$$

The constructed equations from boundary conditions of the problem as they can be seen in Eqs. (58), (59) are limited because we assume the value of $n$ is higher than $m$ earlier in this methodology. But more equations are needed to construct a system of $n+1$ unknowns and $n+1$ equations. So, the remaining equations can be made by substituting Eq. (57) in Eq. (55) as follows:

60 $$\Theta : \left\{\begin{array}{l}\Gamma \left(\zeta \left(0\right),{\zeta }^{\prime}\left(0\right),{\zeta }^{^{\prime\prime}}\left(0\right),\ldots ,{\zeta }^{\left(m-1\right)}\left(0\right)\right)=0 \\ \Gamma \left(\zeta \left(L/2\right),{\zeta }^{\prime}\left(L/2\right),{\zeta }^{^{\prime\prime}}\left(L/2\right),\ldots ,{\zeta }^{\left(m-1\right)}\left(L/2\right)\right)=0\\ \Gamma \left(\zeta \left(L\right),{\zeta }^{\prime}\left(L\right),{\zeta }^{^{\prime\prime}}\left(L\right),\ldots ,{\zeta }^{\left(m-1\right)}\left(L\right)\right)=0\end{array}\right.$$

61 $$\uptheta {^{\prime}}: \left\{\begin{array}{l}\Gamma \left(\zeta ^{\prime}\left(0\right),{\zeta }^{^{\prime\prime}}\left(0\right),{\zeta }^{^{\prime\prime\prime}}\left(0\right),\ldots ,{\zeta }^{\left(m-1\right)}\left(0\right)\right)=0 \\ \Gamma \left(\zeta ^{\prime}\left(L/2\right),{\zeta }^{^{\prime\prime}}\left(L/2\right),{\zeta }^{^{\prime\prime\prime}}\left(L/2\right),\ldots ,{\zeta }^{\left(m-1\right)}\left(L/2\right)\right)=0\\ \Gamma \left(\zeta ^{\prime}\left(L\right),{\zeta }^{^{\prime\prime}}\left(L\right),{\zeta }^{^{\prime\prime\prime}}\left(L\right),\ldots ,{\zeta }^{\left(m-1\right)}\left(L\right)\right)=0\end{array}\right.$$

62 $$\Theta^{\prime\prime\prime}: \left\{\begin{array}{l}\Gamma \left(\zeta ^{\prime\prime}\left(0\right),{\zeta }^{^{\prime\prime\prime}}\left(0\right),{\zeta }^{^{\prime\prime\prime\prime}}\left(0\right),\ldots ,{\zeta }^{\left(m-1\right)}\left(0\right)\right)=0 \\ \Gamma \left(\zeta ^{\prime\prime}\left(L/2\right),{\zeta }^{^{\prime\prime\prime}}\left(L/2\right),{\zeta }^{^{\prime\prime\prime\prime}}\left(L/2\right),\ldots ,{\zeta }^{\left(m-1\right)}\left(L/2\right)\right)=0\\ \Gamma \left(\zeta ^{\prime\prime}\left(L\right),{\zeta }^{^{\prime\prime\prime}}\left(L\right),{\zeta }^{^{\prime\prime\prime\prime}}\left(L\right),\ldots ,{\zeta }^{\left(m-1\right)}\left(L\right)\right)=0\end{array}\right.$$

**Figure 3:**
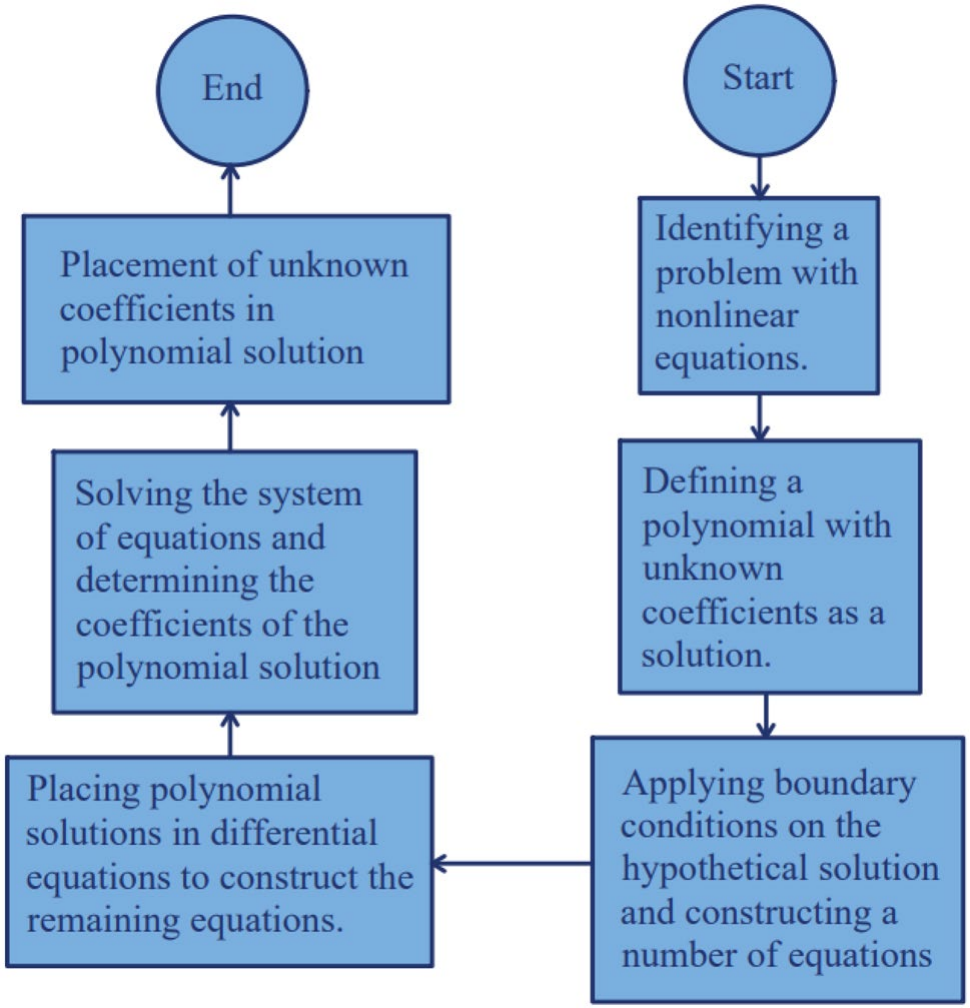
The flow chart of the modified AGM.

So, it can be derived as many equations as possible from Eqs. (60)–(62) to construct a system of $n+1$ unknowns and $n+1$ equations. Finally, series constant coefficients and, thus, the solution to the problem will be determined by solving the equations. Unlike the HAN method, AGM does not depend on the numerical solution and is more independent, but the limitation of this method is that the more nonlinear the problem, the more difficult it is to solve with the AGM method. To summarize the mentioned method in a more compact form, the following Fig. 3, the flow chart is presented for the modified AGM:

### Application of the modified Akbari–Ganji method

In this part, Eqs. (9)–(14) are solved with the modified Akbari–Ganji method for cases (1, 2) according to Table 1. For applying the Modified Akbari–Ganji Method, the following functions are to be assumed the semi-analytical solutions of Eqs. (9)–(14) for cases (1, 2):

63 $$\begin{aligned}&f\left(\upxi \right)=\sum_{i=0}^{5}{a}_{i}{\upxi }^{i} , \mathrm{g}\left(\upxi \right)=\sum_{i=0}^{4}{b}_{i}{\upxi }^{i} , k\left(\upxi \right)=\sum_{i=0}^{4}{c}_{i}{\upxi }^{i} ,\\ & h\left(\upxi \right)=\sum_{i=0}^{4}{d}_{i}{\upxi }^{i},\uptheta \left(\upxi \right)=\sum_{i=0}^{4}{e}_{i}{\upxi }^{i} ,\upphi \left(\upxi \right)=\sum_{i=0}^{4}{w}_{i}{\upxi }^{i} ,\end{aligned}$$

Based on Eq. (63), there are 31 unknown coefficients, and 43 equations are needed to obtain them. Equation (15) makes only 13 equations, and the remaining 18 equations must be made through Eq. (60), and the solutions for cases (1, 2) are as follows:

Solutions of case 1 where $Pr=6.6$, $Nt=0.2$, $Nb=0.2$, $Sc=2.0$, $S=0.0$, $\delta =1.0$ are are demostrated in Eqs. (64)–(69) as follows:

64 $$f\left(\upxi \right)=-0.004692 {\upxi }^{5}+ 0.03247 {\upxi }^{4}- 0.1667 {\upxi }^{3}+ 0.3521 {\upxi }^{2}$$

65 $$\mathrm{g}\left(\upxi \right)=-0.06605 {\upxi }^{4}+ 0.2122 {\upxi }^{3}+ 0.00005 {\upxi }^{2}- 0.3725\upxi + 1$$

66 $$k\left(\upxi \right)=0.004971 {\upxi }^{4}+ 0.02976 {\upxi }^{3}- 0.5 {\upxi }^{2}+ 0.8908\upxi$$

67 $$h\left(\upxi \right)=-0.04149 {\upxi }^{4}+ 0.1308 {\upxi }^{3}- 0.2263\upxi$$

68 $$\uptheta \left(\upxi \right)=0.1507 {\upxi }^{4}- 0.3825 {\upxi }^{3}- 0.3264 {\upxi }^{2}+ 1.558\upxi$$

69 $$\upphi \left(\upxi \right)=-0.2483 {\upxi }^{4}+ 0.3854 {\upxi }^{3}+ 0.3264 {\upxi }^{2}+ 0.5365\upxi$$

Solutions of case 2 where $Pr=6.7$, $Nt=0.4$, $Nb=0.4$, $Sc=4.0$, $S=0.3$, $\delta =1.0$ are are demostrated in Eqs. (70)–(75) as follows:

70 $$f\left(\upxi \right)=-0.01005 {\upxi }^{5}+ 0.05074 {\upxi }^{4}- 0.1667 {\upxi }^{3}+ 0.2961 {\upxi }^{2}$$

71 $$\mathrm{g}\left(\upxi \right)=-0.04035 {\upxi }^{4}+ 0.1255 {\upxi }^{3}+ 0.15 {\upxi }^{2}- 0.515\upxi + 1$$

72 $$k\left(\upxi \right)=-0.01245 {\upxi }^{4}+ 0.06601 {\upxi }^{3}- 0.5 {\upxi }^{2}+ 0.8518\upxi$$

73 $$h\left(\upxi \right)=-0.03891 {\upxi }^{4}+ 0.1186 {\upxi }^{3}- 0.2\upxi$$

74 $$\uptheta \left(\upxi \right)=0.1074 {\upxi }^{4}- 0.1016 {\upxi }^{3}- 0.7832 {\upxi }^{2}+ 1.777\upxi$$

75 $$\upphi \left(\upxi \right)=-0.1824 {\upxi }^{4}- 0.02673 {\upxi }^{3}+ 0.7832 {\upxi }^{2}+ 0.4259\upxi$$

But it can reach more accurate solutions by increasing $n$ in assumed functions. The following functions are the semi-analytical solutions of Eqs. (9)–(14) for cases (3, 4):

76 $$\begin{aligned}&f\left(\upxi \right)=\sum_{i=0}^{8}{a}_{i}{\upxi }^{i} , \mathrm{g}\left(\xi \right)=\sum_{i=0}^{7}{b}_{i}{\upxi }^{i} , k\left(\upxi \right)=\sum_{i=0}^{7}{c}_{i}{\upxi }^{i} ,\\ & h\left(\upxi \right)=\sum_{i=0}^{7}{d}_{i}{\upxi }^{i},\uptheta \left(\upxi \right)=\sum_{i=0}^{7}{e}_{i}{\upxi }^{i} ,\upphi \left(\upxi \right)=\sum_{i=0}^{7}{w}_{i}{\upxi }^{i} ,\end{aligned}$$

Based on Eq. (76), 49 unknown coefficients and 49 equations are needed to obtain them. Since the number of constant coefficients has increased, the number of equations that we must create to make a system of $n$ equations and $n$ unknowns increases, and in addition to Eqs. (60), (61) should also be used. Equation (15) makes only 13 equations, and the remaining 36 equations must be made through Eqs. (60), (61) and the solutions for cases (3, 4) are as follows:

Solutions of case 3 where $Pr=7.1$, $Nt=0.6$, $Nb=0.6$, $Sc=6.0$, $S=0.5$, $\delta =1.0$ are are demostrated in Eqs. (77)–(82) as follows:

77 $$f\left(\upxi \right)=0.00005792 {\upxi }^{8}- 0.0009224 {\upxi }^{7}+ 0.00504 {\upxi }^{6}- 0.02286 {\upxi }^{5}+ 0.06754 {\upxi }^{4}- 0.1667 {\upxi }^{3}+ 0.2655 {\upxi }^{2}$$

78 $$\mathrm{g}\left(\upxi \right)=0.002465 {\upxi }^{7}- 0.01615 {\upxi }^{6}+ 0.0484 {\upxi }^{5}- 0.08819 {\upxi }^{4}+ 0.1006 {\upxi }^{3}+ 0.25 {\upxi }^{2}- 0.6114\upxi + 1$$

79 $$k\left(\upxi \right)=-0.0005115 {\upxi }^{7}+ 0.0004945 {\upxi }^{6}+ 0.00777 {\upxi }^{5}- 0.04093 {\upxi }^{4}+ 0.09967 {\upxi }^{3}- 0.5 {\upxi }^{2}+ 0.8265\upxi$$

80 $$h\left(\upxi \right)=0.0005215 {\upxi }^{7}- 0.005633 {\upxi }^{6}+ 0.02701 {\upxi }^{5}- 0.08334 {\upxi }^{4}+ 0.1377 {\upxi }^{3}- 0.1848\upxi$$

81 $$\uptheta \left(\upxi \right)=0.1268 {\upxi }^{7}- 0.5709 {\upxi }^{6}+ 0.9562 {\upxi }^{5}- 0.7127 {\upxi }^{4}+ 0.546 {\upxi }^{3}- 1.392 {\upxi }^{2}+ 2.047\upxi$$

82 $$\upphi \left(\upxi \right)=-0.287 {\upxi }^{7}+ 1.156 {\upxi }^{6}- 1.51 {\upxi }^{5}+ 0.5827 {\upxi }^{4}- 0.5552 {\upxi }^{3}+ 1.392 {\upxi }^{2}+ 0.2206\upxi$$

Solutions of case 4 where $Pr=7.3$, $Nt=0.8$, $Nb=0.8$, $Sc=8.0$, $S=0.6$, $\delta =1.0$ are demostrated in Eqs. (83)–(88) as follows:

83 $$f\left(\upxi \right)=0.0001189 {\upxi }^{8}- 0.001447 {\upxi }^{7}+ 0.006926 {\upxi }^{6}- 0.02711 {\upxi }^{5}+ 0.07368 {\upxi }^{4}- 0.1667 {\upxi }^{3}+ 0.2522 {\upxi }^{2}$$

84 $$\mathrm{g}\left(\upxi \right)=0.002773 {\upxi }^{7}- 0.01744 {\upxi }^{6}+ 0.05022 {\upxi }^{5}- 0.07932 {\upxi }^{4}+ 0.06954 {\upxi }^{3}+ 0.3 {\upxi }^{2}- 0.6572\upxi + 1$$

85 $$k\left(\upxi \right)=-0.0003986 {\upxi }^{7}- 0.0001867 {\upxi }^{6}+ 0.009358 {\upxi }^{5}- 0.04747 {\upxi }^{4}+ 0.1109 {\upxi }^{3}- 0.5 {\upxi }^{2}+ 0.8142\upxi$$

86 $$h\left(\upxi \right)=0.0006773 {\upxi }^{7}- 0.006581 {\upxi }^{6}+ 0.02953 {\upxi }^{5}- 0.08572 {\upxi }^{4}+ 0.1357 {\upxi }^{3}- 0.1771\upxi$$

87 $$\uptheta \left(\upxi \right)=0.1783 {\upxi }^{7}- 0.7272 {\upxi }^{6}+ 1.134 {\upxi }^{5}- 1.014 {\upxi }^{4}+ 1.279 {\upxi }^{3}- 2.238 {\upxi }^{2}+ 2.389\upxi$$

88 $$\upphi \left(\upxi \right)=-0.4353 {\upxi }^{7}+ 1.59 {\upxi }^{6}- 1.846 {\upxi }^{5}+ 0.7763 {\upxi }^{4}- 1.276 {\upxi }^{3}+ 2.238 {\upxi }^{2}- 0.04631\upxi$$

## Results and discussion

**Figure 4:**
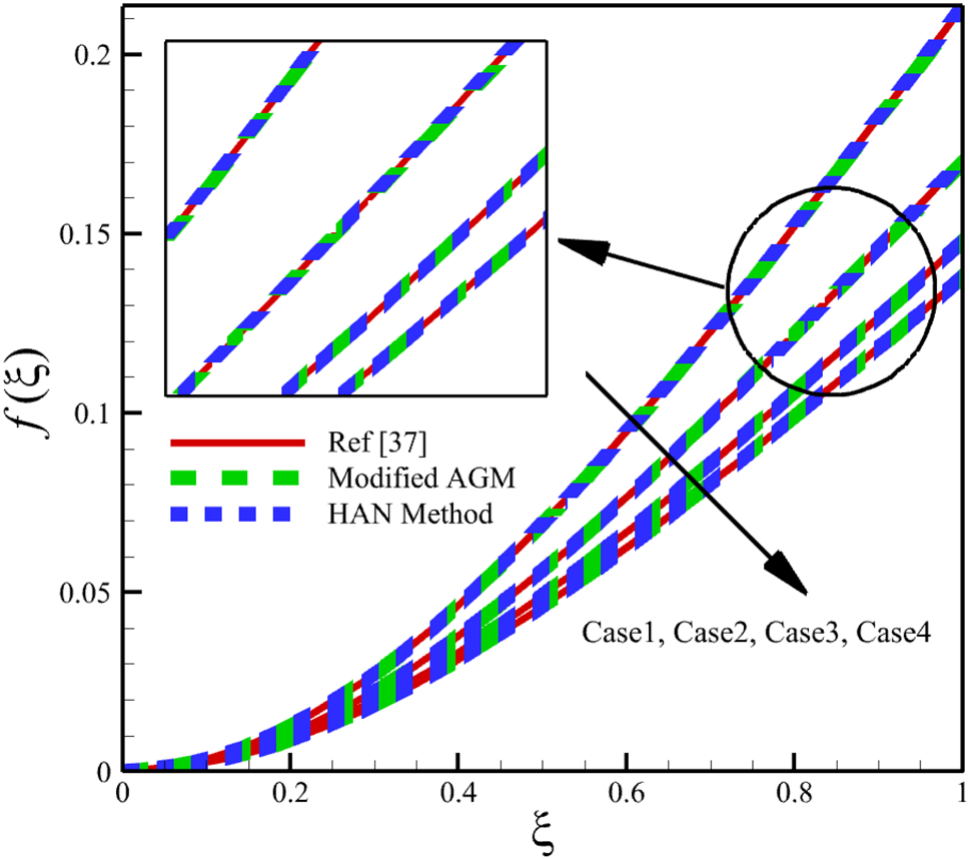
The impact of different cases on $f\left(\upxi \right)$.

**Figure 5:**
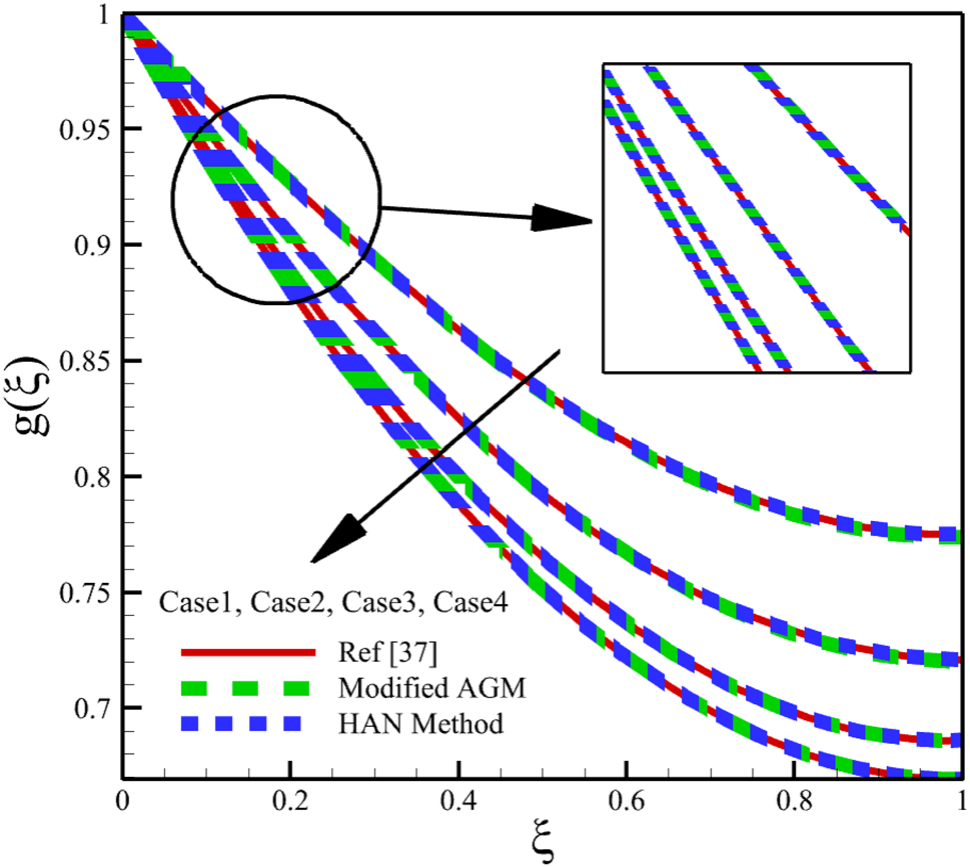
The impact of different cases on $\mathrm{g}\left(\upxi \right)$.

**Figure 6:**
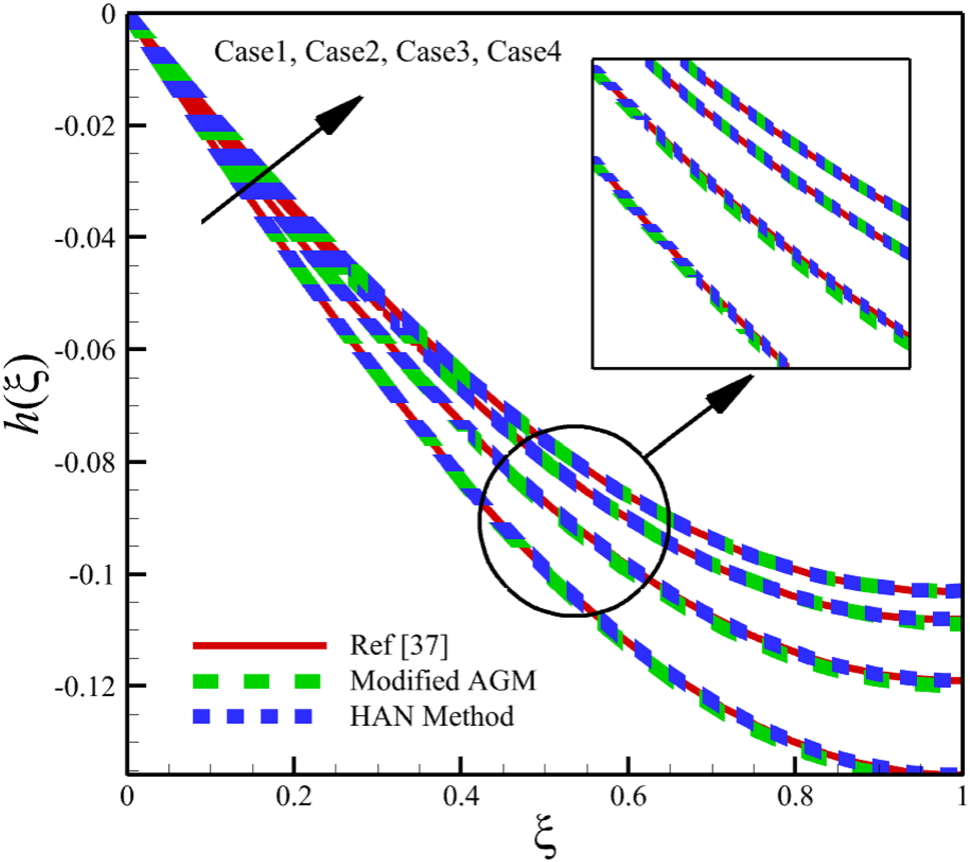
The impact of different cases on $h\left(\upxi \right)$.

**Figure 7:**
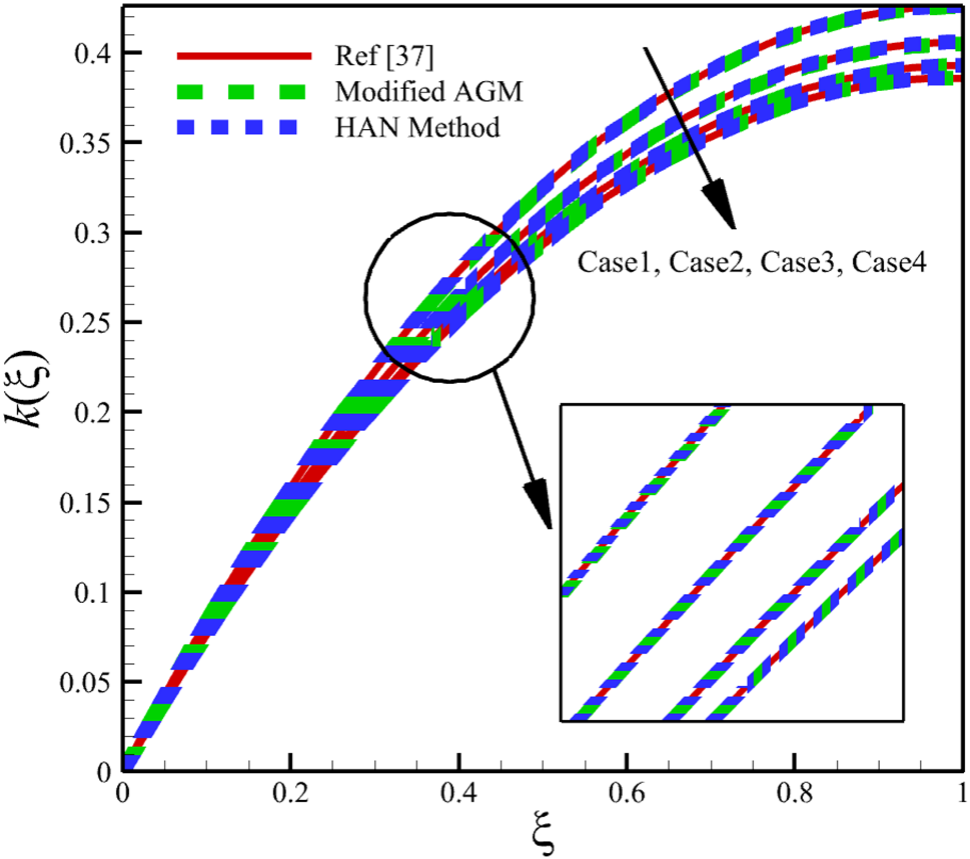
The impact of different cases on $k\left(\upxi \right)$.

**Figure 8:**
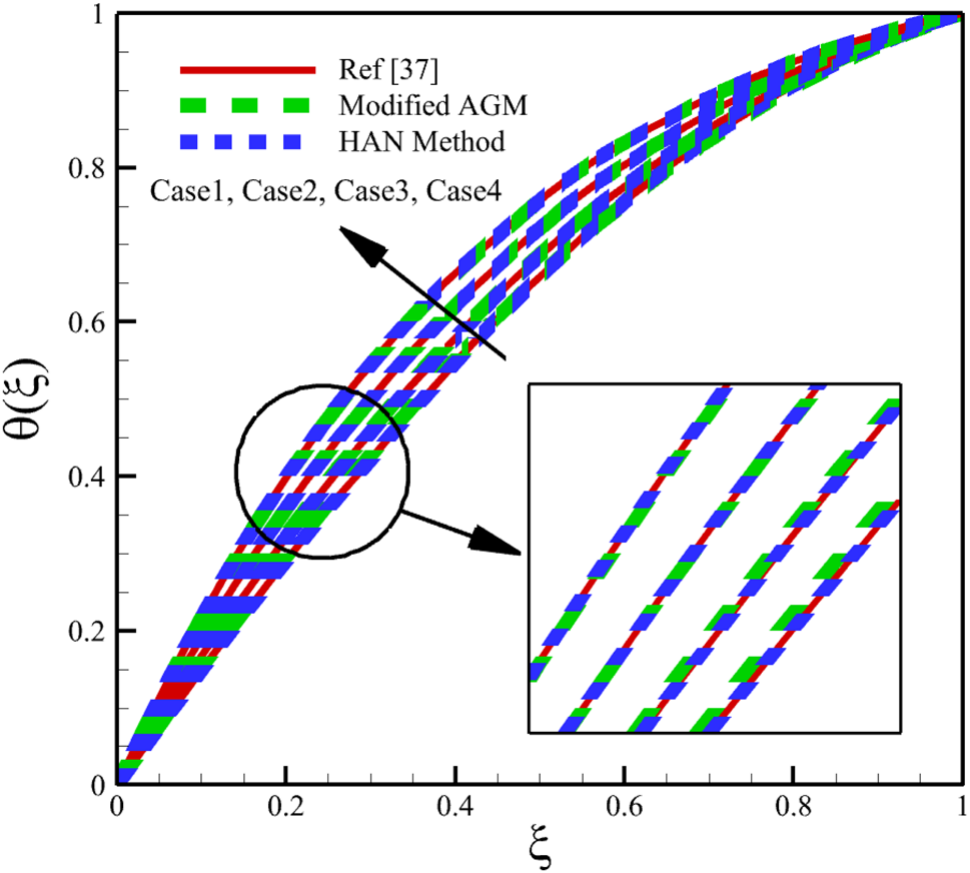
The impact of different cases on $\uptheta \left(\upxi \right)$.

**Figure 9:**
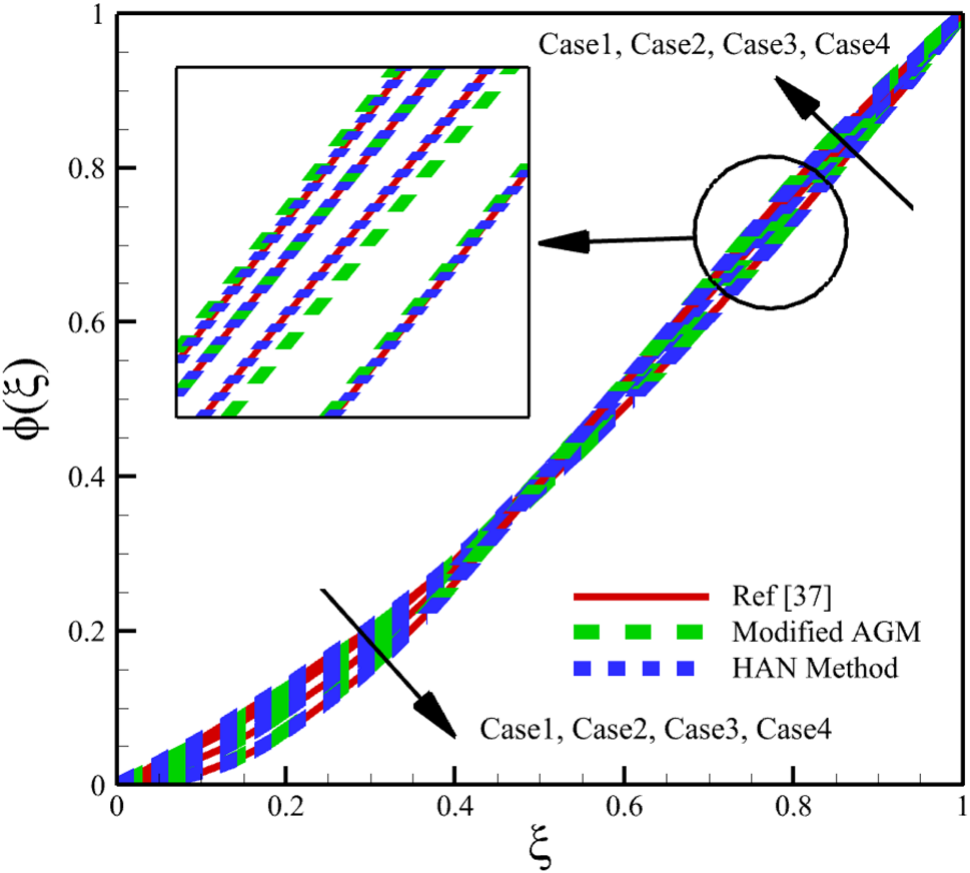
The impact of different cases on $\upphi \left(\upxi \right)$.

**Figure 10:**
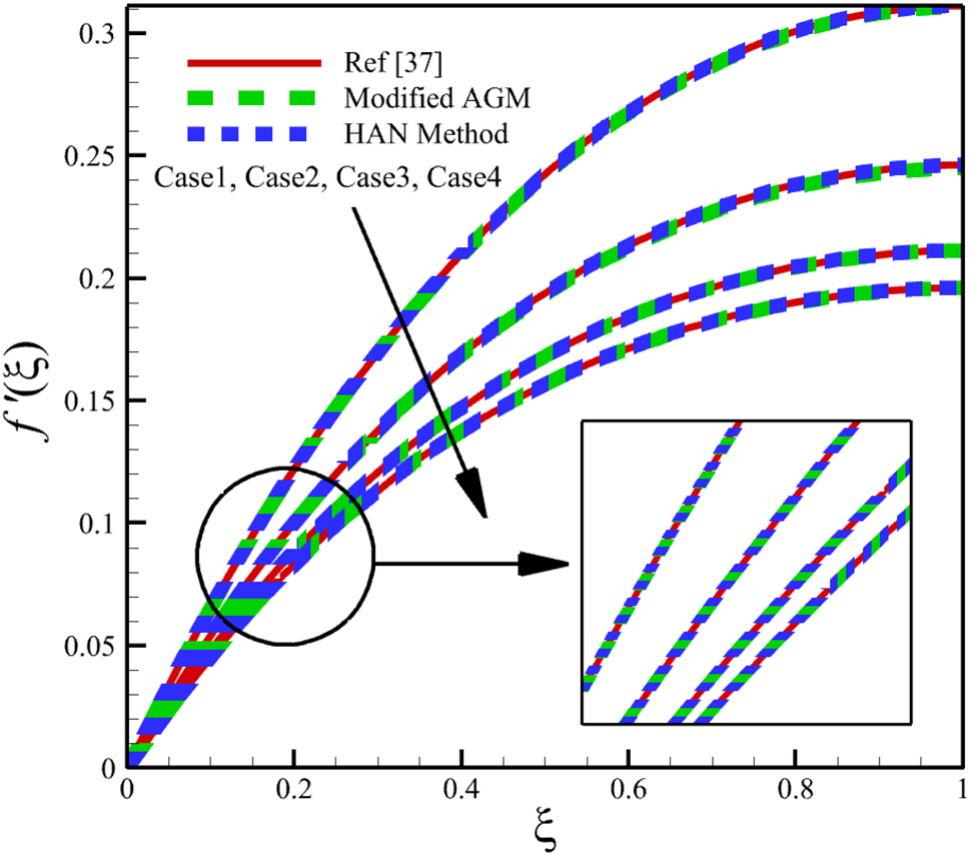
The impact of different cases on $f^{\prime}\left(\upxi \right)$.

**Figure 11:**
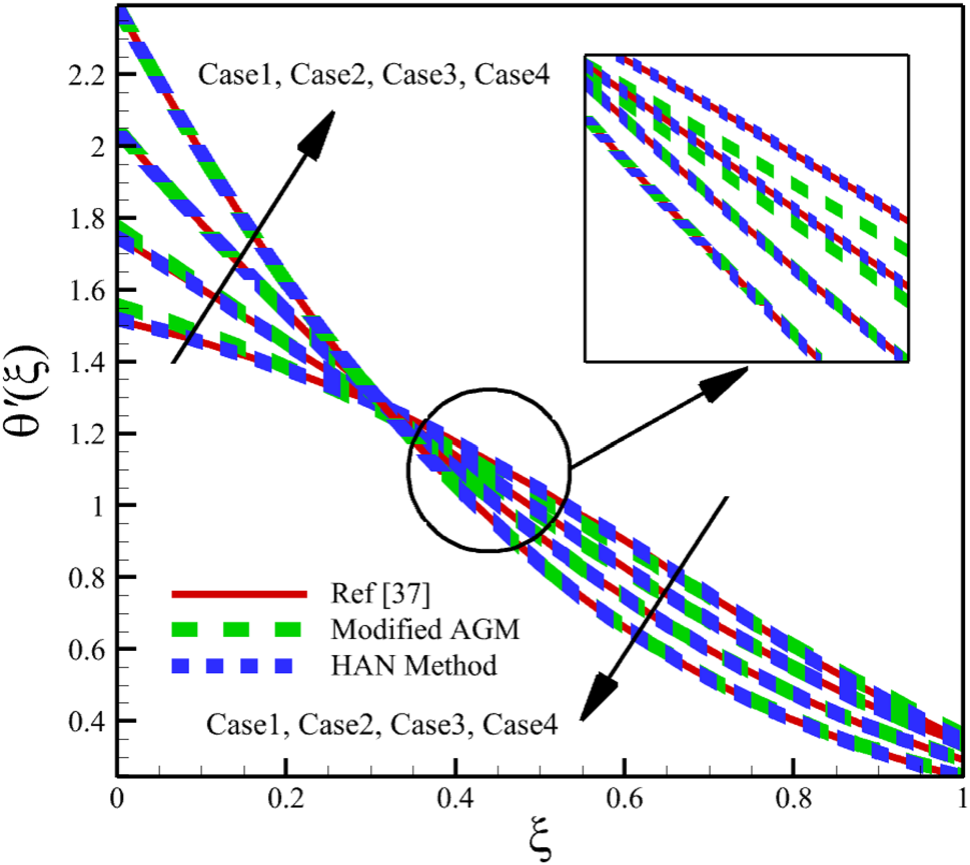
The impact of different cases on $\uptheta ^{\prime}\left(\upxi \right)$.

**Figure 12:**
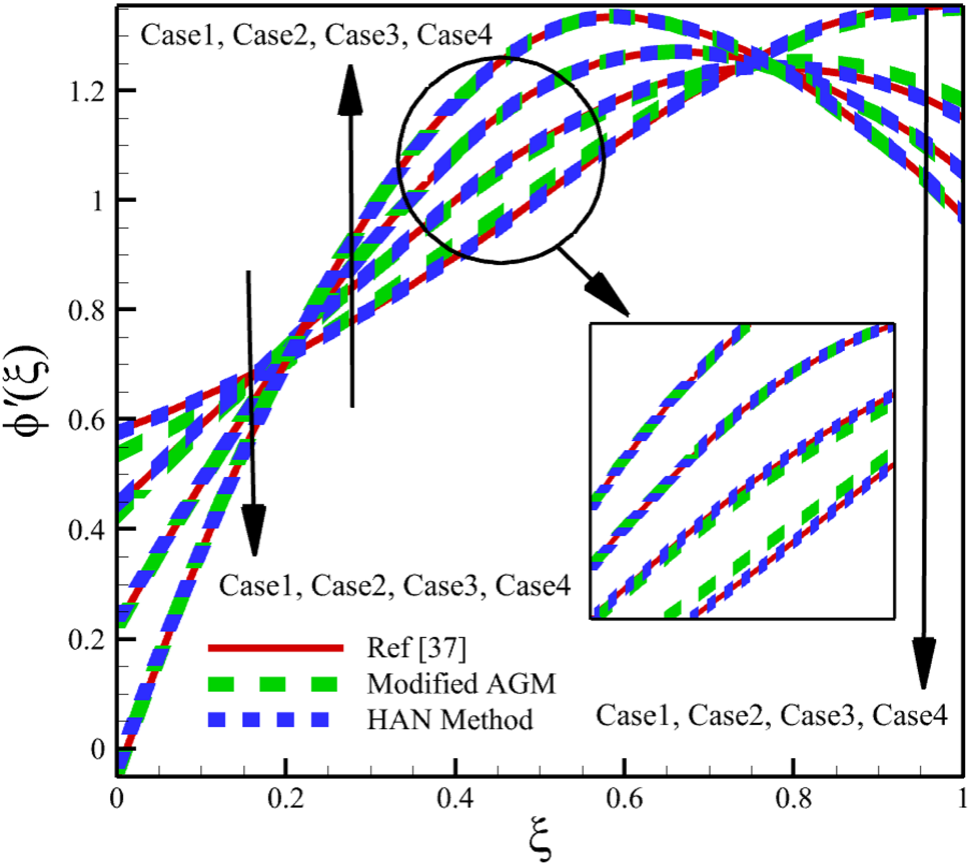
The impact of different cases on $\upphi ^{\prime}\left(\upxi \right)$.

Heat and mass transfer in an unsteady rotating inclined plane has been investigated using 3D thin film nanomaterials flow. The solutions were obtained using the modified AGM and HAN methods. These two analytical solutions' validity was proved when compared with the Zeeshan et al.^37^ Runge–Kutta fourth-order (RK4) numerical solutions. Figures 4, 5, 6, 7, 8, 9, 10, 11 and 12 show the accuracy of the modified AGM and HAN results. The following table relates to the four cases considered in this study.

As the impact of four cases is shown in Figs. 4, 5, 6, 7, 8, 9 and variations of Sherwood number, heat transmission, and radial velocity profiles are illustrated in Figs. 10, 11 and 12. In this study, the average value of some arbitrary scalar function of $\upchi \left(\upxi \right)$, is defined as follows:

89 $${\overline{\upchi } }_{\mathrm{avg}}=\sum_{i=a}^{b}\frac{\upchi \left({\upxi }_{\mathrm{i}}\right)}{b-a+1} ,$$

where the $a$ and $b$ are integer numbers. According to Eq. (89), the average values of $f\left(\upxi \right)$, $f^{\prime}\left(\upxi \right)$,$\mathrm{g}\left(\upxi \right)$, $k\left(\upxi \right)$, $h\left(\upxi \right)$, $\uptheta \left(\upxi \right)$, $\upphi \left(\upxi \right)$, $\uptheta {^{\prime}}\left(\upxi \right)$, and $\upphi{ ^{\prime}}\left(\upxi \right)$ are denoted by ${\overline{f} }_{avg}$, ${\overline{f} }_{avg}^{\prime}$,${\overline{\mathrm{g}} }_{avg}$, ${\overline{k} }_{avg}$, ${\overline{h} }_{avg}$, ${\overline{\uptheta } }_{avg}$, ${\overline{\upphi } }_{avg}$, ${\overline{\uptheta } }_{avg}^{\prime}$, and ${\overline{\upphi } }_{avg}^{\prime}$ , respectively. According to Figs. 4, 5, 6, 7, 8, 9, 10, 11 and 12, when cases (1–4) related to Table 1 occur, the average values of ${\overline{f} }_{avg}$, ${\overline{f} }_{avg}^{\prime}$, ${\overline{\mathrm{g}} }_{avg}$, ${\overline{k} }_{avg}$, ${\overline{h} }_{avg}$, ${\overline{\uptheta } }_{avg}$, ${\overline{\upphi } }_{avg}$, ${\overline{\uptheta } }_{avg}^{\prime}$, and ${\overline{\upphi } }_{avg}^{\prime}$ , are given in the following tables:

In Tables 2, 3 and 4, some average results increased when the conditions changed from case 1 to case 4, and some decreased. The decrease or increase of these values is calculated using the following relationship:

90 $$\Omega =\frac{{\mathrm{ Z}}_{2}-{\mathrm{ Z}}_{1}}{\left|{\mathrm{ Z}}_{1}\right|}\times 100,$$

where $\Omega$ is the amount of percentage increase or decrease values, ${\mathrm{ Z}}_{2}$ is the second value and ${\mathrm{ Z}}_{1}$ is the first value. According to Table 2, when the constant coefficients of cases in Table 1 change from case 1 to case 4, the average values of the results from Ref.^37^ change respectively. When the conditions change from case 1 to case 4, ${\overline{f} }_{avg}$ , will decrease by 34.71362167%, ${\overline{f} }_{avg}^{\prime}$ will decrease by 35.78465385%, ${\overline{\mathrm{g}} }_{avg}$, will decrease by 8.197818397%, ${\overline{k} }_{avg}$ will decrease by 9.504907967%, ${\overline{h} }_{avg}$ will increase by 23.38976383%, ${\overline{\uptheta } }_{avg}$ will increase by 9.441723369%, ${\overline{\uptheta } }_{avg}^{\prime}$ will increase by 7.742384880%, and ${\overline{\upphi } }_{avg}^{\prime}$ will decrease by 9.974419462% but according to Table 2, ${\overline{\upphi } }_{avg}$ will increase by 1.541136126% when it changes from case 1 to case 2 but, ${\overline{\upphi } }_{avg}$ , will decrease by 1.839033024% when it changes from case 2 to case 4. According to Table 3, when the constant coefficients of cases in Table 1 change from case 1 to case 4, the average values of the results from Modified AGM change respectively. When the conditions change from case 1 to case 4, ${\overline{f} }_{avg}$ , will decrease by 34.60665497%, ${\overline{f} }_{avg}^{\prime}$ will decrease by 35.68188954%, ${\overline{\mathrm{g}} }_{avg}$ , will decrease by 8.140379566%, ${\overline{k} }_{avg}$ will decrease by 9.388178325%, ${\overline{h} }_{avg}$ will increase by 23.82760391%, ${\overline{\uptheta } }_{avg}$ will increase by 9.259738964%, ${\overline{\uptheta } }_{avg}^{\prime}$ will increase by 7.367258115%, and ${\overline{\upphi } }_{avg}^{\prime}$ will decrease by 9.528160070% but according to Table 2, ${\overline{\upphi } }_{avg}$ will increase by 1.313715539% when it changes from case 1 to case 2 but, ${\overline{\upphi } }_{avg}$ , will decrease by 1.109559162% when it changes from case 2 to case 4. According to Table 4, when the constant coefficients of cases in Table 1 change from case 1 to case 4, the average values of the results from the HAN Method change respectively. When the conditions change from case 1 to case 4, ${\overline{f} }_{avg}$ , will decrease by 34.71362166%, ${\overline{f} }_{avg}^{\prime}$ will decrease by 35.78461613%, ${\overline{\mathrm{g}} }_{avg}$ , will decrease by 8.197818386%, ${\overline{k} }_{avg}$ will decrease by 9.504907931%, ${\overline{h} }_{avg}$ will increase by 23.38976385%, ${\overline{\uptheta } }_{avg}$ will increase by 9.441723405%, ${\overline{\uptheta } }_{avg}^{\prime}$ will increase by 7.592028101%, and ${\overline{\upphi } }_{avg}^{\prime}$ will decrease by 9.661365531% but according to Table 2, ${\overline{\upphi } }_{avg}$ will increase by 1.541136126% when it changes from case 1 to case 2 but, ${\overline{\upphi } }_{avg}$ , will decrease by 1.839033024% when it changes from case 2 to case 4.

**Table 2:** The average values of the results from Ref.^37^ in different cases.

Average results	Case 1	Case 2	Case3	Case 4
${\overline{f} }_{avg}$	0.08661520073	0.06960507141	0.06051673148	0.05654792764
${\overline{f} }_{avg}^{\prime}$	0.2019348372	0.1610516327	0.1392084656	0.1296731547
${\overline{\mathrm{g}} }_{avg}$	0.8607268700	0.8249151010	0.8015399316	0.7901660443
${\overline{k} }_{avg}$	0.2726459024	0.2595413164	0.2509559060	0.2467311603
${\overline{h} }_{avg}$	− 0.08423992761	− 0.07395768082	− 0.06756432786	− 0.06453640749
${\overline{\uptheta } }_{avg}$	0.5831929114	0.5979073648	0.6176815710	0.6382563728
${\overline{\upphi } }_{avg}$	0.4401036474	0.4468862437	0.4436788908	0.4386678581
${\overline{\uptheta } }_{avg}^{\prime}$	0.9872590589	1.008966158	1.033950988	1.063696455
${\overline{\upphi } }_{avg}^{\prime}$	0.9926099707	0.9598263023	0.9285715314	0.8936028886

**Table 3:** The average values of results from Modified AGM in different cases.

Average results	Case 1	Case 2	Case3	Case 4
${\overline{f} }_{avg}$	0.08646285600	0.06943472266	0.06050127950	0.05654095375
${\overline{f} }_{avg}^{\prime}$	0.2015693760	0.1604994000	0.1391575116	0.1296456139
${\overline{\mathrm{g}} }_{avg}$	0.8601848800	0.8246159600	0.8015207172	0.7901625658
${\overline{k} }_{avg}$	0.2722924290	0.2591193866	0.2509684823	0.2467291302
${\overline{h} }_{avg}$	− 0.08474165600	− 0.07457810400	− 0.06759786558	− 0.06454974986
${\overline{\uptheta } }_{avg}$	0.5839460800	0.5989518933	0.6176979840	0.6380179627
${\overline{\upphi } }_{avg}$	0.4387271467	0.4444907734	0.4437344534	0.4395588853
${\overline{\uptheta } }_{avg}^{\prime}$	0.9916900000	1.010920000	1.034355883	1.064750362
${\overline{\upphi } }_{avg}^{\prime}$	0.9888800000	0.9608170000	0.9273398934	0.8946579307

**Table 4:** The average values of results from the HAN Method in different cases.

Average results	Case 1	Case 2	Case3	Case 4
${\overline{f} }_{avg}$	0.08661520072	0.06960507141	0.06051673148	0.05654792764
${\overline{f} }_{avg}^{\prime}$	0.2019347938	0.1610516188	0.1392084894	0.1296732030
${\overline{\mathrm{g}} }_{avg}$	0.8607268700	0.8249151010	0.8015399316	0.7901660444
${\overline{k} }_{avg}$	0.2726459024	0.2595413165	0.2509559059	0.2467311604
${\overline{h} }_{avg}$	− 0.08423992760	− 0.07395768083	− 0.06756432789	− 0.06453640747
${\overline{\uptheta } }_{avg}$	0.5831929113	0.5979073646	0.6176815709	0.6382563729
${\overline{\upphi } }_{avg}$	0.4401036474	0.4468862437	0.4436788906	0.4386678581
${\overline{\uptheta } }_{avg}^{\prime}$	0.9877082408	1.008678113	1.033180332	1.062695328
${\overline{\upphi } }_{avg}^{\prime}$	0.9920285129	0.9605227917	0.9303649948	0.8961850121

## Conclusion

This study investigates the problem of heat and mass transfer in an unsteady rotating inclined plane using 3D thin film nanomaterial flow. The governing equations were set PDEs, and by using suitable similarity transformation, the PDEs were reduced into a set of nonlinear ODEs. The ODEs in four cases were solved with two semi-analytical techniques of Modified AGM and HAN. The Modified AGM that is used in this study is a novel technique, and the novelty of current work is related to solving this problem analytically. Unlike the former AGM, the Modified Agm has solved the previous issues and can replace the previous method of AGM. The HAN Method is another semi-analytical method that transforms a numerical solution into an analytical one. Technically, if the numerical solution exists for some problem, then HAN Method can be applied to obtain an analytic solution. The results of the HAN solution are very close to the Numerical solutions of Zeeshan et al.^37^ when compared with the modified AGM, but at the same time, the modified AGM is not dependent on any numerical methods for approximating analytical solutions. So, this paper is concluded that:

- A new semi-analytical is introduced by modifying the former AGM technique.
- The exact analytic solutions were obtained through HAN Method.
- The solutions of both analytical solutions were compared with previously published papers.
- The results of both analytical solutions were presented quantitatively.
- The Sherwood number of the film surface and inclined swirling surface will decrease as the Schmidt number increases and the angular velocity of the rotating surface decreases.
- The Nusselt number of inclined swirling surfaces will increase as the Prandtl number increases and the angular velocity of the rotating surface decreases.
- The Nusselt number of film surfaces will decrease as the Prandtl number increases and the angular velocity of the rotating surface decreases.

## Data Availability

The datasets used and/or analysed during the current study available from the corresponding author on reasonable request.
